# Giant primary cystic mediastinal lymphangioma: A case report

**DOI:** 10.3892/ol.2014.2320

**Published:** 2014-07-04

**Authors:** BINGJUN YANG, CHUNYANG JIANG, BAOQIN ZHANG, QI REN, TAO TANG, SHENG XU, HONGRUI XU, HONG YAO, YOUKUI HAN, SHUZHONG LIU, LI LI, HUI ZHAO

**Affiliations:** 1 Department of Thoracic Surgery, Tianjin Union Medicine Centre, Tianjin 300121, P.R. China; 2 Department of Gastroenterology, The Fifth Center Hospital of Tianjin City, Tianjin 300450, P.R. China; 3 School of Public Health, Hebei United University, Tangshan, Hebei 063001, P.R. China; 4 Department of Pathology, Tianjin Union Medicine Centre, Tianjin 300121, P.R. China; 5 Department of Neurosurgery, Tangshan People’s Hospital, Tangshan, Hebei 063001, P.R. China

**Keywords:** cystic mediastinal lymphangioma, surgery, recurrence

## Abstract

Cystic lymphangioma mainly occurs in children. Cystic mediastinal lymphangioma (CML) originates from mediastinal tissues and is an extremely uncommon cystic lymphangioma that develops from the lymphatic vessels. The present study reports the case of 46-year-old male patient with a giant CML that was surgically resected by video-assisted thoracoscopy. The largest diameter of the CML was 18.0 cm, and ~400 ml of pale yellow fluid was removed from the cystic cavity during surgery. The postoperative pathological reports on the cystic wall showed that the neoplasm was a CML. At present, at the one-year postoperative follow-up, there are no signs of recurrence. In conclusion, complete surgical resection may prevent recurrence.

## Introduction

Cystic lymphangioma, also known as cystic hygroma, is a congenital malformation originating from lymphatic hyperplasia. Cystic lymphangioma is a type of hamartoma and verges on the clinical boundary between tumor and deformity. The majority of lymphangiomas are observed in patients under the age of five (1), with extremely few cases reported in adulthood. This disease can occur in various areas of the body, with the most common location being the neck. Usually, the tumors are slow-growing, with an asymptomatic clinical course. Cystic lymphangiomas are commonly soft and painless masses, but cannot easily be compressed.

Cystic mediastinal lymphangioma (CML) is an extremely uncommon benign cystic lymphangioma developed from the lymphatic vessels. With regard to cystic lymphangioma, only ~1% are mediastinal (2). CMLs are most often located in the anterior mediastinum. In order to improve the diagnosis and treatment of CML in clinical practice, knowledge on the topic must be compiled and shared. The present study reports the case of a giant anterior CML for this purpose. Patient provided written informed consent.

## Case report

**Figure 1:**
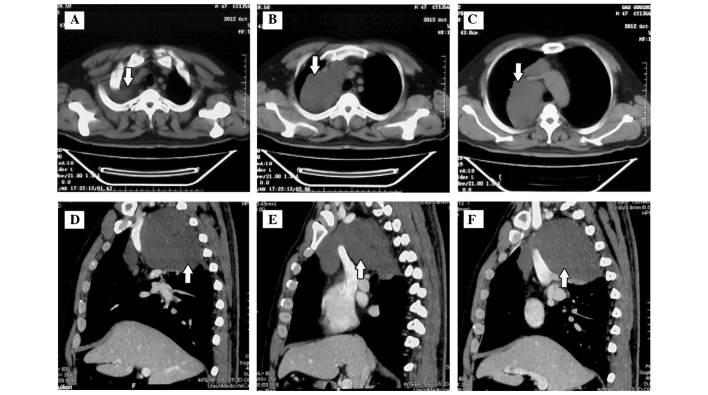
Thoracic computed tomography images of the mediastinal tumor. (A-C) Thoracic cross-sectional imaging showing the different layers of the tumor. The lesion is indicated by downward-pointing arrows. (D-F) Thoracic vertical section imaging showing the layers of the tumor, indicated by upward-pointing arrows.

A 46-year-old, male, non-smoker was diagnosed with a right anterior mediastinal tumor by computed tomography (CT) scan during a physical examination in October 2012 (Fig. 1). No significant previous medical history was reported and no specific clinical manifestations. Examinations performed prior to surgery included test of pulmonary function and narrow band imaging bronchoscopy, and no abnormal findings were observed. The results of the analysis for tuberculosis (TB) antibody and TB-DNA in the serum were all negative. Serum tumor markers for lung carcinoma, including carcinoembryonic antigen, carbohydrate antigen-125 (CA-125), squamous cell carcinoma (SCC), CA72-4, cytokeratin 19 fragments, neuron-specific enolase and ferritin were all within the normal ranges. The initial tentative diagnosis was of a thymoma or bronchocele.

**Figure 2:**
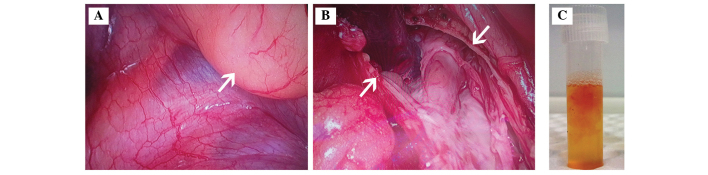
Representative images of the surgery and liquid sample obained. (A) The mediastinal tumor observed by video-assisted thoracoscopy prior to resection. The surface of the mass is indicated by the white arrow. (B) The surgical field observed by video-assisted thoracoscopy following removal of the mass; the border is indicated by the white arrows. (C) The pale yellow liquid absorbed from the cystic cavity and collected in a tube.

**Figure 3:**
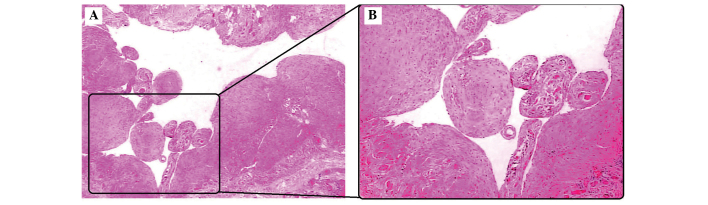
Pathological results of the mediastinal tumor. Following hematoxylin and eosin staining, the histopathological changes of the resected cystic wall of the tumor were observed by light microscopy and images were captured. Representative images of the post-operative pathological results are shown. (A) The multilocular cystic cavity in the cystic wall tissues surrounded by smooth muscle and lymphoid tissue (magnification, ×20). (B) Histopathological tissue from the black-bordered box in (A) presented at increased magnification (magnification, ×40).

Subsequently, the lung resection of the mediastinal tumor was performed by video-assisted thoracoscopy. A cystic and globose tumor, with the largest diameter of 18.0 cm, was located in the lateral section of the right anterior mediastinum (Fig. 2A). Following separation of the cyst wall from the base, the cystic wall was removed (Fig. 2B). During the surgery, ~400 ml of pale yellow liquid was absorbed from the cystic cavity (Fig. 2C). Following resection of the cystic wall and hematoxylin-eosin staining, the histopathology was observed under a light microscope (Nikon Eclipse 80i; Nikon, Tokyo, Japan). The postoperative pathological examination of the cystic wall showed multilocular cystic cavity in the cystic wall, surrounded by smooth muscle and lymphoid tissue, as well as the neoplasm. As a result, a diagnosis of CML was determined (Fig. 3). At the one-year follow-up there were no signs of recurrence.

## Discussion

Mainly occurring in childhood, cystic lymphangiomas are extremely rare, with 90% being diagnosed prior to two years of age. A limited number of studies exist with regard to cystic lymphangioma in adults (3). CML is an extremely rare vascular tumor originating from the lymphatic vessels. In total, <1% of cystic lymphangiomas occur in the mediastinum and >90% are discovered in individuals under two years old (4). CML is benign and is usually an incidental finding unless there are symptoms caused by compression of local tissues and structures or infection. The CT images of CML often resemble adenopathy or a mass. For adult patients, the probable diagnosis would be of a thymoma, bronchocele or malignancy.

Complete resection may be difficult in certain cases due to their proximity to vital structures in the mediastinum (5). Although other treatment methods, such as sclerotherapy and radiotherapy, have been reported in unresectable cases, they are generally ineffective and may result in hemorrhage and infection (6). Therefore, surgery remains the superior method for treatment with curative intent. Complete surgical resection remains the treatment of choice for lymphangioma in order to eliminate symptoms and prevent recurrences (7). The risk of tumor recurrence due to an incomplete excision ranges between 0 and 13.6%, while the aspiration of cystic fluid only decreases cyst size for a short time and introduces the patient to the risk of infection (8). Currently, there are few documented cases of giant CML (9–11). In the present study, the largest diameter of the CML was 18.0 cm and the volume removed from the cystic cavity during surgery was ~400 ml. Histological analysis, the gold standard method, was able to confirm the CML diagnosis.

In summary, CML, particularly giant CML, is extremely rare in adults. Complete surgical resection provides a definitive histological diagnosis and prevents recurrence.
